# Deconstructing (e)health literacy: aspects that promote and inhibit understanding of health information in breast cancer patient pathways

**DOI:** 10.1080/17482631.2022.2137961

**Published:** 2022-10-21

**Authors:** Heidi Gilstad, Kari Sand, Marit Solbjør, Line Melby

**Affiliations:** a Centre for Academic and Professional Communication, NTNU- Norwegian University of Science and Technology, Norway; b SINTEF Digital, Norway; c Department of Public Health and Nursing, NTNU, Norway

**Keywords:** Cancer patient pathway, breast cancer, information and communication, health literacy, eHealth literacy, health information, deconstructing health literacy, understanding

## Abstract

Purpose Deconstructing current definitions of “health literacy (HL)” and “eHealth literacy (eHL)”, into the core notion of “understanding health information (HI)”, this study provides insights into what promotes and inhibits the understanding of HI for breast cancer patients during cancer patient pathways (CCP) in Norway. Methods Seven well-educated women were interviewed. Through a stepwise deductive-inductive analysis of the transcribed interviews, the following topics were identified: 1) explanations accompanied by drawings, 2) individualized knowledge-based information, 3) information processing capacity, and 4) ambiguity in medical information. Results The women's understanding of HI increased when spoken communication was accompanied by visual illustrations, which served as roadmaps throughout the CPP. Even if HI should be targeted to the patients’ individual needs, some HI can be generalized if it refers to established knowledge about the health phenomena. The women described their changing mental and physical status during the CPP and how these changes influenced their understanding of HI. Conclusion The results challenge the idea that HL and eHL are fixed, stable, personal characteristics. On the contrary, HL/eHL, in this case particularly the understanding of HI, depends on the individual (temporary) physical and cognitive capacity of the patient and adaptation in the institutional and private contexts.

## Introduction

Breast cancer is the most prevalent cancer worldwide. In 2020, 2.3 million women were diagnosed with breast cancer and 685,000 died of breast cancer globally (World Health Organization [WHO], 2021). Becoming a patient involves not just life changes and existential issues related to identity and health, but also practical issues, such as time and economy. For adequate help, the patient must find how the healthcare system works and which professionals to approach about different questions. Breast cancer patients report a need for information and communication about several issues concerning their condition and medical treatment (Brattheim et al., 2017), with needs varying through the pathway from the first suspicion of cancer to post-treatment care. Trustworthy information from reliable sources may help overcome the emergent need for knowledge.

In Norway, all patients who present symptoms that are likely to be cancer are referred to a standardized cancer patient pathway (CPP). CPP has been an organizing principle for the standardized care of cancer patients since 2015 (Melby et al., 2021). The aim of patient pathways is to ensure that cancer patients encounter a well-organized, integrated, and predictable pathway, without any unnecessary non-medically justified delays in investigation, diagnosis, treatment, and rehabilitation (helsenorge.no). CPP comprise 3 phases: Time from suspecting cancer to first appointment within specialist health services, time between diagnostic procedures and confirmed diagnosis, and waiting time from having a diagnosis until the start of treatment. The service is designed to be patient-centred, and the patient must be seen and treated individually, if possible, accompanied by next-of kin. CPP ensures the predictability of timeframes from suspicion to diagnosis (Melby & Håland, 2021). However, a Danish study found that although the CPPs were predictable in terms of time and organization, they were inadequate in terms of information, communication, involvement in treatment choices, flexibility, and access to the clinic after surgery (Løwe et al., 2020). A Norwegian study showed that patients found it difficult to take part in shared decision-making during the CPP, as most of their decisions relied on professional advice (Andersen-Hollekim et al., 2021). A premise for being able to take part in decision-making is that the patient understands the information communicated throughout the CPP.

Provision of information does not mean that the patient understands and acts upon the information. Patients may be functional analphabets or highly literate about the topic, and the degree of health literacy—and eHealth literacy in particular—varies throughout the population (Holt et al., 2019; Simpson et al., 2020; Villadsen et al., 2020). In fact, 33% of the Norwegian population lacks the basic skills to be able to relate to and use health information to maintain their own health (Le et al., 2021). Diverse levels of (e-)health literacy could influence how breast cancer patients perceive information given to them through their cancer patient pathway.

For the patient to be able to cope with her diagnosis and condition and to be able to participate in decision-making, she needs knowledge and skills to identify, critically evaluate, and understand the information provided throughout the pathway. Given that much information today is provided digitally, she needs to be both health- and eHealth literate. But what does it mean to be health literate (HL) or eHealth literate (eHL)? Are HL and eHL stable characteristics in the person?

HL and eHL are fuzzy concepts, embracing several aspects of being a knowledge-seeking citizen or patient. As Pleasant and Kuruvilla (2008) note, the health literacy notion covers both the communication in clinical care, and public health information. To capture the essence of these concepts, let us therefore deconstruct and reinterpret two prominent definitions of HL and eHL, respectively from Nutbeam and Kickbusch (1998), Norman and Skinner (2006), and Nutbeam and Kickbusch (1998) stated that “Health literacy represents the cognitive and social skills which determine the motivation and ability of individuals to gain access to, understand, and use information in ways which promote and maintain good health». Understanding health information is particularly important in the definition.

Nutbeam (2000) elaborated on the notion, and distinguished broadly between the three levels of health literacy: basic/functional literacy (sufficient basic skills in reading and writing to be able to function effectively in everyday situations), communicative/ interactive literacy (more advanced cognitive and literacy skills that, together with social skills, can be used to actively participate in everyday activities, extract information and derive meaning from different forms of communication, and to apply new information to changing circumstances) and critical literacy (more advanced cognitive skills that, together with social skills, can be applied to critically analyse information and to use this information to exert greater control over life events and situations).

Although not explaining explicitly what “understanding” is, Nutbeams distinction into three levels of health literacy is interesting because of its inclusion of communicative and contextual dimensions. The distinction presupposes the actively information-seeking citizen. Likewise, when defining eHL, the psychologists Norman and Skinner (2006) suggested that eHL is “the ability to seek, find, understand, and appraise health information from electronic sources and apply the knowledge gained to addressing or solving a health problem.” Understanding is again a core in the definition but related to information in electronic sources. The notion “understanding” is implicit in the mentioned definition, but is elaborated in the Lily model, where the authors illustrated that eHealth literacy’s fundamental components were traditional literacy and numeracy, science literacy, health literacy, information literacy, media literacy, and computer literacy. Also in this model, the notion of understanding is part of the literacy concept, without being specifically explained.

The eHL concept of Norman and Skinner has been criticized for not considering contextual and communicative aspects (Gilstad, 2014), and has been redefined and elaborated in adjustment with socio-technological changes over time (Norman, 2011). New definitions of eHealth literacy have been offered, often accompanied by elaborated measurement instruments (Griebel et al., 2018; Lars et al., 2018; Norgaard et al., 2015; Paige Samantha et al., 2017; Van der Vaart & Drossaert, 2017). Although being useful for mapping tendencies on a population level, such measurement instruments (often based on self-reporting) are problematic because they rest upon the idea that HL and eHL are stable, measurable entities.

In this paper, we take a different approach. We assume that there are several aspects that promote and inhibit HL and eHL, especially the understanding of health information. Measurement of HL for a healthy citizen is different from when measuring a newly diagnosed cancer patient. Likewise, measuring at one point in the patient pathway will assumingly be significantly different than measuring the same way later in the trajectory. This study investigates qualitatively how breast cancer patients experienced communication and information exchange with health care personnel throughout the CPP. Our research question is:

Which aspects promote and inhibit the understanding of health information and communication for breast cancer patients during cancer patient pathways (CCP)?

Transparency is a key word in qualitative studies. After the methods section, we describe analytical considerations and choices. We then conduct the analysis, focusing on four main themes: 1) explanations accompanied by drawings, 2) individualized knowledge-based information, 3) information processing capacity, and 4) ambiguity in medical information.

## Methods

The methodological underpinnings for the data collection and analysis are socio-constructivist, with an aim to examine how events, meanings, and experiences are discursive constructions in society. Realities are social constructions, and are subjective and multiple (Berger & Luckmann, 1967). Language is more than mere reflections of reality. We “do things with words,” and with language (Austin, 2003). The assumption is that reality is constituted through discourse, and the individual experiences and meaning-making inform, and are informed, by discourses in their local or broader contexts. Interactionism is a perspective on language and communication with roots in socio-constructivism. In an interactionist perspective, discourse is relational, and a joint construction between the participants (Linell, 1998). This occurs in everyday conversations, but also in more professional conversation, such as the research interview. The interviewer and the participant produce the narrative about the participants experiences, opinions, perspectives etc. in a co-construction (Gubrium & Holstein, 2016). The interviewer is a co-producer of knowledge, since she is present during the interview, explicitly leads the participant into topics through the questions in the interview guide and influencing the dialogue through the way she responds to the participants communicative contributions. In the interactionist perspective, data is created throughout the conversation, and is not giving direct knowledge of something that has happened. The interviewee will only report her *interpretation* of what happened, and her thoughts, feelings, emotions, reflections on the matter. There may be several interpretations of the same event. As interviewers, we need to be conscious about how we actively influence the narrative that is being created (Gubrium & Holstein, 2016).

To capture the patients’ reported experiences, we adopted a retrospective design and conducted in-depth interviews with seven participants (n = 7), recruited through a patient organization. Two of the researchers (author 1 and 2) contacted the patient organization and asked if the Facebook page administrator was willing to inform about the study on Facebook. The researchers also informed about the study during an event at a centre for support and recreation for cancer patients, survivors, and relatives. Patients or survivors contacted the researchers by email if interested in taking part in the study, and appointments for interviews were scheduled. Written informed consent was obtained when the participants and the researchers met.

The inclusion criterion was that the participant had been diagnosed with breast cancer after the CPPs’ introduction, and consequently, had experienced a CPP.

Seven ethnic Norwegian women with higher education, aged between 45 and 65 years old, were interviewed. They were diagnosed with breast cancer 1–4 years before the time of the interviews. Four of the participants said that they were diagnosed about two years ago. The interviews lasted between 45 to 100 minutes.

An interview guide was developed, and the following topics were addressed through the interview: present health situation, information received by the health system, experiences with communication with health care professionals, and experiences with information search. During user tests, the participants were asked to show the researcher what they would do if they had to use a computer to find information about CPP for breast cancer.

Two researchers (author 1 and 2 of this paper) were present during the interviews The researchers have PhDs in respectively language and communication studies and medicine and are trained in research interviewing. Both have MAs in applied linguistics, and have worked as health communication researchers (on different topics, including cancer) at a university hospital and in R&D. During the interviews, one of the researchers asked the questions and guided the participants’ attention and conversation, and the other researcher made notes. The interviews were audio-recorded. Immediately after the interviews, the researchers discussed and summarized the conversations. The audio recordings were transcribed orthographically. The notes and transcripts were the primary materials of the study.

This study was approved by the Norwegian Centre for Research Data and the internal review board at the Cancer Clinic, St. Olavs hospital, Trondheim University Hospital.

## Analytical approach

In all qualitative approaches, clarity on the process and practice of how the research is conducted is particularly important (Braun & Clarke, 2006). In this project we identify, analyse and report on four topics derived from coding and categorizing our interview data.

The analysis of the verbatim transcribed interviews was conducted through a nitty-gritty detailed analysis of the transcripts of the interviews. The interview transcripts were coded with a stepwise inductive-deductive approach (SDI; Tjora, 2021), an iterative process of reading the transcripts, making notes, and identifying the focal topics related to communication and information exchange. The interview is a speech event, where the interviewer and the interviewed co-create the discourse (Mishler, 1986) Although semi structured, the main topics in the interview guide triggered a wide range of associations in the participants. In interviews of patients, this is common (Brinkmann, 2014). The interviewers also allowed for free association from the participants, as they considered it unethical to interrupt them in the middle of their narratives of sensitive topics. The participants referred to different communication situations involving different persons and contextual characteristics. Because of the density of topics started by the participants in the interviews, the coding process was complicated. Following the SDI (Tjora, 2021) we did narrow readings of the empirical data, using concepts that appeared in the transcripts. In the sections of the transcripts, we drew out codes, that is, significant units of words and utterances. This was an inductive process, and the first part of the analysis, though still more descriptive than analytic. Based on a collection of these codes, and drawing on the research question, we defined categories. This is also an inductive process, intricately linked to the data, but is at the same time deductive, as it refers to the predefined patterns formulated in the research questions.

The patients accounts included many aspects about their understanding of health information during the CPP. Consequently, departing from the deconstructed definitions of HL and eHL (see above), we found units and sequences about their understanding of HI throughout the pathway (Table I).

**Table I.** What promotes and inhibits the understanding of health information (HI)?

What promotes understanding of HI?	What inhibits understanding of HI?
Physician’s appearance/ personality/ method of communication Trust in information source Trust in professional healthcare worker Time/ space opportunity/ repetitions/ to process information The doctor’s information method, visualizing the information during the conversation Training/ advice on how to get information That health professionals take the time to explain Easy access to health professionals Talking to others who had cancer Network with peers Co-reading information with peers To get to know as much as possible about the body and health status Facilitation of information and learning Rehabilitation and treatment coordination Need for various sources of information spoken, written, multimodal Need for timely and individually adapted information	Cognitive impairment Emotional distress Pervading sense of crisis Concentration problems due to chemotherapy Mentally influenced by bad news Divergence between diagnosis and physical lack of pain Inhibitory memories of the place you were told about the diagnosis Negative messages become bodily experiences Impaired memory Not to be told what you need to know The burden of having to inform others Word choice with unfortunate/ non-constructive associations Information adaption to the actual health service Burden (time, worry) of searching information Assuming an active patient role

The table illustrates the coded topics concerning respectively what promotes and what inhibits understanding of health information. The women were not explicitly asked what promoted and inhibited their understanding but reflected more broadly on their experiences with information and communication in the CPP-process.

The analysis is an interpretative process, and in this project performed primarily as an inductive, empirically driven identification of topics and interpretation, considering existing research on the topic. We acknowledge that the four categories discussed here are limited, and our aim is not to provide generalizable results. The analysis of interview data cannot make claims about how the breast cancer patients understand health information in general. On the contrary, we point to a few particular phenomena concerning what promotes and inhibits understanding of HI.

The insights from this study may contribute to the knowledge development about breast cancer patients’ reflections based on experiences concerning health information in the CPP. We also hope that these insights may be a small contribution to the discourses about definitions and the measurement instruments of HL and eHL.

## Analysis

The analysis is organized with two main questions: What promotes understanding of health information? and What inhibits understanding of health information? More specifically, we shed light on the following topics: 1) explanations accompanied by drawings, 2) individualized knowledge-based information, 3) information processing capacity, and 4) ambiguity in medical information. In the analysis, we use authentic examples from the anonymized interviews. For anonymization, we apply excerpts without recognizable markers.

## What promotes understanding of health information?

### Explanations accompanied by drawings

The health care personnel’s strategies for informing about health has consequences for how the patient understands her diagnosis. A communicative strategy often applied by healthcare professionals when talking to patients, regardless of their individual literacy level, is to draw sketches to illustrate the points they are making verbally (Osborne, 2006). In the following example, we see how the participant reflects upon the significance of the medical explanations and evaluations accompanied by the drawing:

Excerpt 1.

**Table:** 

1	Participant:	On October 23—exactly one month after the operation—I went to the oncologist, and he did something very, very fundamentally important to me.
2		He drew on a sheet of paper my receptors, the tumour, and explained to me what all the receptors meant, how big—what was good, and what was not good.
3		[…]
4		HER2 was negative for my tumour, and it was a violent cancer … I have grade 3 [cancer]; it is aggressive.
5		All of this he explained, and then wrote it down … he turned the sheet over, and then wrote down my course of treatment. I was going to go on EC90, the first chemotherapy (onomatopoeic icon), for three weeks—every three weeks. Then, I was going to go on Taxana, that is, Taxotere or Taxol, for three four 12 weeks, and then there was radiation.
6		What happened to the immune system in relation to the risk of infection, fever, and how I should deal with it?
7	Interviewer:	Yes.
8	Participant:	Many prescriptions for many drugs that I should take … a regime …
9	Interviewer 2:	So, he took the time to write it down, instead of saying it?
10	Participant:	Yes, on a sheet of paper.
11		[…]
12		And he managed to do all that—even give me a requisition for a wig. He somehow managed to run the whole race in such a controlled and calm way, and then I got that sheet with me.
13	Interviewer:	Yes, brought it home and …
14	Participant:	It’s like a roadmap … where you in a way have something to lean on.

In Excerpt 1, the participant reflects on the communicative strategies of the oncologist during a consultation in the CPP one month after the operation. According to the participant, the oncologist first *drew* on a sheet of paper. The drawing consisted of anatomical details (“receptors”, “tumour”) concerning the condition of this particular patient. Secondly, the oncologist *explained* what the drawing represented. According to the participants´ report, he *described* the anatomy (“how big”), and he *evaluated* the anatomic details for the particular patient “what was good, and what was not good”(utterance 2). He subsequently *wrote* comments to the drawing, and he *wrote the “course of treatment”*. In this detailed explanation by the interview participant, we even learn that the oncologist “turned the sheet over”, indicating that the first page was full of drawed information. The information about the course of treatment consisted of medication (“Taxana, that is Taxotere or Taxol”), therapy (“chemotherapy” and “radiation”), as well as indications of time (“for three weeks-every three weeks”). The interview participant reported that in his explanation, the oncologist *informed about risk* (“what happened to the immune system in relation to the risk of infection”). In relation to risk issues, he also *gave advise* about how the patient behave (“how should I deal with it”). All the communicative activities emphasized above, drawing, explaining, describing, evaluating, writing, advising, and informing about medication, time and risk issues, were done with a pragmatic adaptation of personalized and generalized knowledge.

The participant signalled that she felt the information was personalized, by her use of nouns “my receptors” (utterance 2), “my tumour” (utterance 4), “my course of treatment” (utterance 5), “how I should deal with it” (utterance 6), “drugs that I should take” (utterance 8). However, the information conveyed from the oncologist was knowledge-based (for examples issues concerning anatomy, diagnostic assessment and decisions, prognosis, risk, preferred or advised treatment for the specific diagnostic decision etc), and part of the professional practice and conduct of an oncologist. Based on the reporting on the interviewed participant, we do not know what words the oncologist used, but some of the words and acronyms the participant uses when reporting are clearly medical (“receptors”, “HER2”, “EC90”).

The oncologist made a drawing, and then verbally explained to the patient about her medical condition, course of treatment, and medication. The drawing became a significant artefact for the patients’ understanding of her diagnosis. Moreover, it represented a remembering-aid she could lean on when she came home. It served as a “road map,” and a support for her in accepting diagnosis and treatment. The written text and the drawing provided information that could be transferred to a setting outside the hospital.

Visual illustrations are powerful tools for communication for several reasons (Lee et al., 2021). They help understand phenomena and the relationship among them. They also can help display difficult scientific terms and words. What we see in the example above is that drawing also helps in remembering. In her account in the interviews of the communication with the doctor, the informant used medical terms and pharmaceutics names, thus displaying what she had learned about what she needed to know and which steps to take.

Professionals communicating with potentially vulnerable persons in healthcare settings need the ability to take the position of “the other” (Goffman, 1959), and adjust information to an adequate level. This other orientation includes the ability to empathize with the other. In the literature on health literacy, there is a tendency to focus on patients that, for varied reasons, are marginalized from society because of limited understanding (Villadsen et al., 2020). As illustrated in the following quote, the need for information adjusted to ones’ level of knowledge and preferences, also apply for highly literate patients who prefer advanced information:

Excerpt 2.

**Table:** 

1	Participant	I think he was very good and took his time too… [compared with] the surgeon.
2		He immediately understood. He adjusted when he talked to me, and
3		sometimes maybe he wrote the word or the term … when he thought I was
4		not involved. Then, I stopped and asked what he meant, and then he
5		explained it too. He was very good at adjusting to the patient’s knowledge.

As shown in Excerpt 2, the patient appreciated that, in the consultation, the oncologist spent time to communicate with her. According to the participant, the oncologist “immediately understood” (utterance 2), referring to his understanding of her well-informed questions. The participant in the above example had a university degree in life sciences. In the interview, she explained that she read what she came across in the research literature about her condition, and she was not afraid of Latin terms or chemical formulas. She had high scientific literacy and critical health literacy (Nutbeam, 2000), which clarified her health condition to herself. Nevertheless, she needed to communicate with an expert, and to verify whether what she had read and understood aligned with the knowledge of the oncologist. The participant reported that the oncologist “adjusted” (utterance 2) verbally to her level of understanding. Moreover, he *wrote* the words and terms (utterance 3). This participant reflects explicitly on the communicative exchange between her and the oncologist, and how he adjusted to her involvement (utterance 4) and her understanding (“I stopped and asked what he meant”), and provided explanations adjusted to the patient’s knowledge. This communicative adjustment seems to have satisfied her need for verifying knowledge-based, generalized information, and at the same time it reassured her personally regarding insecurity concerning her cancer diagnosis.

## Individualized knowledge-based information

Holistic, collaborative, and responsive care, while simultaneously adjusting to the individual patient is an important focus in the patient-centred paradigm (Sidani & Fox, 2014). However, one participant was critical to the approach that everything is relative owing to the individual differences among people. According to her, communication with patients and services must include more generalized knowledge-based information.

Excerpt 3.

**Table:** 

1	Participant:	Ah! That word! The phrase that makes me most disgusted is “people are different.”
2		This is not true. Then, they can avoid saying anything because people are so different.
3		People are not very [expletive] different. Everyone I have talked to, has been like me in many ways. Some are like that, and some are like that, but …
4	Interviewer2:	So, if you say, “people are different,” then you have somehow covered everything instead of saying that-?
5	Participant:	Yes, then you say nothing. […] Yes, it’s like they reject the topic in a way,
6		and then I think “who am I, then?”
7		The same applies to side effects as well. They cannot say anything about side effects because people are very different.
8		Alternatively, they should not give much information because people are overwhelmed by it.

The participant in this excerpt reflected on personalized health information-giving due to the individual differences between patients, which the patient-centred paradigm in healthcare is based upon. She did not agree to the saying that “people are so different” (utterance 1). On the contrary, she claimed that people are not that different (utterance 3), and that people, and in this case patients, have many similarities: “Everyone I have talked to, has been like me in many ways (utterance 3). Encouraged by the follow-up question by interviewer 2 (utterance 4), which indicates a direction for the further reasoning, the participant suggests that healthcare professional “reject the topic” (utterance 5). Not receiving knowledge-based, generalized information challenges her: “who am I, then” (utterance 6), indicating that not disclosing information gives the patient a sense of not being taken seriously. The participant particularly addresses information of risk of side effects from treatment (utterance 7).

The participant in this example insisted that some information must be generalizable, and that health care personnel do not always need to try to adjust to the individual on a topic level. On the contrary, they should treat patients similarly and provide the same information, be that the diagnosis or on possible side effects of treatment. Diagnostics and treatment procedures, although constantly developing, are knowledge-based, and often other patients have experienced the same medical phenomena. This participant argues that health care personnel should inform about medical issues, but also clearly frame information-giving in a way to open for interruptions from the patient if they do not find it understandable or relevant. With her utterance “Who am I then?” she signals the profoundly ethical issue of wanting to be taken seriously by the health care personnel, by telling her facts, and not trying to cover the seriousness of the health diagnosis and treatment behind utterances that may mislead the patient to believing that this may or may not happen to her.

This participant was a health professional by profession and had a hybrid professional and patient approach to communication about breast cancer. She had many explicit examples of how health professionals can communicate with cancer patients. The example above illustrates the communicative dilemmas of professional conduct in the frames of the overall patient-centred paradigm, where they must balance communicatively between the patients demand for personalized information, but at the same time maintaining the ethical and professional duty to communicate knowledge-based information.

## What inhibits the understanding of health information?

### Information processing capacity due to treatment side effects

Cancer patients report about cognitive problems, bad memory, and difficulties in the mental capacity to read and learn after cancer treatment (Von Ah & Crouch, 2020). In our study, several participants reflected the lack of energy resulting from diagnosis and treatment. One of the participants told that during chemotherapy, her ability to concentrate and memorize as well as her ability to handle practical issues related to e.g., her job and sick leave, were highly reduced. At one point she was so tired that she had a death wish:

Excerpt 4.

**Table:** 

1	Participant:	I have accepted and been through such phases where I am terrified to die, and then I had a phase where I was
		—No, you know, now I am so tired … of being afraid to die … that now I can just as well die.
		Then, I wanted to die, but I did not die. Then, I wanted to die, but I did not die.

The excerpt is about an important *premise* for being able to perceive and understand health information. When a person is concerned with a death wish because of physical and mental side effects of diagnosis and treatment, the capacity to process health information may be challenged. Treatment includes side effects that may influence the patient’s understanding of health information. Because of the lack of cognitive ability, mental distress, fatigue, and depression, searching for information, reading, and understanding health information is difficult. The example illustrates that cognitive skills are not permanent entities but are changeable and influenced by how they are regarded and talked about in the context and depending on the condition and treatment regime of the patient. Two of the patients described that after being informed about their diagnosis they felt like going into a shock, and consequently not being able to absorb any information at all. One of them remembered specifically that this shock phase was temporary. It was over after surgery, “because then I was able to make jokes again”. These experiences illustrate how the ability to understand information may vary during the pathway.

## Ambiguity in medical information

Previously we saw that a participant preferred to receive knowledge-based information during the consultation with the healthcare professionals. However, communicating and understanding knowledge-based information is not straight-forward since information may be complicated and ambiguous. An example is risk information. Risk information is challenging for many reasons (Sarangi & Clarke, 2002). Our study participants expected to receive relevant information in the consultation, including the calculated and expected risks and problems. Determining diagnosis is often a complicated task, and health care personnel cannot always be 100% certain. Moreover, patients may react differently to the treatment, so the outcome of the treatment cannot be 100% predictable. However, as we see in the next example, the participant prefers to receive information about potential future problems to be mentally prepared.

Excerpt 5.

**Table:** 

1	Participant	Maybe it is a prejudice I have—but I think [the medical personnel] believe that if we get to know how bad it can be, then we will not take the treatment. While I believe that, if we find out how bad it will be, then we can prepare.
2		[…]
3		Then, we know that if we do not [suffer] what we could have, it is a bonus. We will just be happy.

Excerpt 5 highlights another the participants reflections about the dilemma of receiving information about treatment and potential problems, with the consequence that the patient may chose not to accept the treatment. The participant assumes that healthcare professionals “believe that if we get to know how bad it can be, then we will not take the treatment” (Utterance 1). The participant, however, believes that “if we find out how bad it will be, then we can prepare”. The participant argues that not informing thoroughly about the risk of potential problems and side effects but offering the treatment for the “uninformed” patient and waiting to see how she reacts to it may be problematic, since the patient will not be able to prepare for eventual challenges. With adequate information, understanding of risk may occur, and consequently it may help the patient to adjust and prepare.

Communication about risks in general and the side effects of medication and treatment are complicated. Healthcare professionals have a legal and moral duty to inform patients with facts, while at the same time reassure, maintain dignity, and give hope (Sarangi, 2017). There are several dilemmas in informing patients of side effects. Information on the risk and severity may contribute to patients not wanting the treatment or the specific medication. Patients experienced that doctors could be uncertain about whether the medication had the desired effect. Despite the rich and highly advanced research and technology in oncology, cancer treatment is still fraught with numerous uncertainties, including what causes side effects and why some side effects in one patient do not appear in others. Finally, the benefit of the medication may nevertheless be worth the possible side effects, irrespective of the unpleasantness of the treatment.

## Results and discussion

Deconstructing the HL and eHL concepts, in this paper we focused the research question: what promotes and inhibits the understanding of health information throughout the CPP.

The women who were interviewed in this project had their existence changed, from being regular citizens to becoming patients with breast cancer who were enrolled in a CPP in the Norwegian healthcare system. When talking to the researchers, they reflected on the challenges with communication and information, particularly on how language, communication, and literacy depend on the cognitive and emotional ability related to the biomedical condition and the overwhelming feelings caused by the total situation of being a patient with a life-threatening disease. Consequently, the need for clear communication adjusted to each patients’ information needs, level of knowledge and preferences, may help their understanding of their health condition. As shown, the main contextual factor inhibiting understanding identified in this study, was the cognitive and emotional alterations caused that made it difficult for the patients to remember, understand or cope with health information. Further, these cognitive and emotional obstacles for understanding were not stable throughout the pathway. This means that it is not only the patients’ information needs that varies throughout the disease trajectory (Brattheim et al., 2017; Goerling et al., 2020), but also contextual factors influencing the patients’ ability to understand information. When health care personnel adjust their information to the patients’ needs and preferences, they face a rather complicated task since the information cannot be provided in the same manner each time due to the mentioned variations. Assessing a patients literacy level in patient-physician communication is different than assessing a population’s literacy level by use of self-reported measurements.

In their efforts of adjusting information to each patient’s needs and preferences, health care personnel may find it difficult to give precise patient-centred information about for instance, side effects of treatment or at what time a patient could expect to go back at work. Due to different outcomes for each patient, it may feel safer to tell a patient that no specific answers can be provided, because future events to a large degree from patient to patient. However, as shown in our analysis, some patients prefer to be given general information instead of no information. General information, or information about what usually happens to patients in a similar situation, could give the patient a feeling of belonging to a group of individuals that matter, and a possibility to prepare for future adverse events.

Visual and textual artefacts accompanying the verbal explanations, evaluations, advice, and adjusted health language were presented as successful in this study and might be useful throughout the disease trajectory despite variations in the patients’ information needs. As shown, the visualizations were not only useful for understanding information, but also for remembering as well as serving a more overall function as road map that provides predictability and safety even at home between the consultations with health care personnel. In a German interview study among patients with breast, colorectal or prostate cancer, it was shown how the patients used information to gain or regain control in a seemingly uncontrollable situation by e.g., understanding the consequences of the disease and treatment for one’s life and dealing with fear (Blödt et al., 2018).

Mishel (1988) noted that patients construct meaning of their illness, and uncertainty occurs when they are unable to find that meaning. Understanding what is being communicated about the health status is crucial for meaning making. This meaning-making may be helped when participants understand what is going on, what the consequences are, and what rights they must take part in the setting to clarify what they do not understand. The question of how and what we can understand has been debated by philosophers for years, with issues such as -understanding is based on experience and what is perceivable- (empiricists) versus -the key to understanding is rational thinking- (rationalism). As Linell (2011, p16) proposes: «Understanding can in principle never fully be made explicit”. We cannot know how others understand concepts and phenomena. Within the limitations of this paper, we have pointed out that both subjective and contextual aspects influence the understanding of health information. Understanding may happen on a *linguistic level*, concerning words, terms, and abstract phenomena, on a *relational level*, such as who to turn to, and on an *institutional level*, such as where to present oneself. Understanding is both a mental capacity and a physical experience. To understand is not the same as to act upon what you understand. You may understand that smoking is dangerous, but still continue smoking.

Consequently, patients’ health- and eHealth literacy levels cannot be easily assessed. As seen in this study, there are various communicative, cognitive, and emotional aspects influencing the understanding of health communication and information through a patient pathway and in the aftermath of the treatment.

These insights may be of value for health professionals who want to adjust their communication with the breast cancer patients. This insight may also have consequences for how researchers and health professionals assess health- and eHealth literacy. Traditional assessment methods, such as questionnaires and simple self-reporting assessment tools, have significant shortcomings, as they do not consider the cognitive and emotional aspects that inhibit health literacy, or the communicative and contextual aspects that may promote health literacy, if conducted adequately.

## Conclusion

The women in this project reflected upon several communicative activities and strategies that are important for their understanding of health communication. First, health communication should be adjusted iteratively to the person during the conversation, regardless of high or low literacy. However, some information can be generalized if it is based on evidence. It is considered helpful for patients´ understanding if spoken communication is accompanied by visual illustrations of the diagnosis, treatment, and medication. Patients appreciate dialogue with professional experts, preferably the same ones throughout the patient pathway. When in a communication situation, it may be difficult to know about the patient’s capability to handle, therefore it may be a wise strategy to be explicit about openness and limitations in conversation, that is, what the health personnel will communicate and what the patient must ask.

## Limitations of the study

This study would have benefited from including a larger and more diverse participant group. All participants were white, well-educated women recruited from the same geographic area.
